# Enrichment of low abundance DNA/RNA by oligonucleotide-clicked iron oxide nanoparticles

**DOI:** 10.1038/s41598-021-92376-9

**Published:** 2021-06-22

**Authors:** Fereshte Damavandi, Weiwei Wang, Wei-Zheng Shen, Sibel Cetinel, Tracy Jordan, Juan Jovel, Carlo Montemagno, Gane Ka-Shu Wong

**Affiliations:** 1 Ingenuity Lab, 1-070C, 11421 Saskatchewan Drive NW, Edmonton, AB T6G 2M9 Canada; 2 Department of Chemical and Materials Engineering, University of Alberta, Edmonton, AB T6G 2V4 Canada; 3 Department of Medicine, University of Alberta, Edmonton, AB T6G 2E1 Canada; 4 Department of Biological Sciences, University of Alberta, Edmonton, AB T6G 2E9 Canada; 5 Present Address: Geneis Inc., Bldg A, 5 Guangshun North Street, Beijing, China; 6 Present Address: Nanotechnology Research and Application Center (SUNUM), Sabanci University, Istanbul, 34956 Turkey

**Keywords:** Genomic analysis, Isolation, separation and purification, Sequencing

## Abstract

Detection of low abundance target DNA/RNA for clinical or research purposes is challenging because the target sequences can be hidden under a large background of human genomic or non-human metagenomic sequences. We describe a probe-based capture method to enrich for target sequences with DNA-clicked iron oxide nanoparticles. Our method was tested against commercial capture assays using streptavidin beads, on a set of probes derived from a common genotype of the hepatitis C virus. We showed that our method is more specific and sensitive, most likely due to the combination of an inert silica coating and a high density of DNA probes clicked to the nanoparticles. This facilitates target capture below the limits of detection for TaqMan qPCR, and we believe that this method has the potential to transform management of infectious diseases.

## Introduction

In many samples that are sequenced for clinical or research purposes, the targets of interest are found in mixtures of asymmetrical abundance, i.e. the target sequences are obscured under a massive background of human genomic or non-human metagenomic sequences. Next generation sequencing (NGS) is effective for surveying medium-to-high abundance targets, but it is more problematic for low abundance targets, e.g. viruses and bacteria at the initial pre-symptomatic or the later chronic stages of infection, non-abundant viruses and bacteria in dirty metagenomics samples, or subsets of gene transcripts that are expressed at low levels. Several approaches have been developed for target enrichment, most notably PCR pre-amplification^1^ and nucleic acids capture^2^. Initial efforts that combined hybridization capture with NGS were onerous, expensive, and time consuming. For instance, the first human gene capture arrays used solid support and expensive instruments like the MAUI hybridization station (NimbleGen). They had run times of 65 h^3,4^. Magnetic particles for separation of nucleic acids have also been developed^5^, a system referred to as solution hybridization capture. DNA probes are biotinylated to enable the isolation of complementary molecules by capturing biotin with high-affinity streptavidin-coated paramagnetic beads^6^.

The coding regions of the human genome (the exome) comprise about 1% of the genome and have arguably been the paramount subject of study for hybridization-based capture and NGS^6–10^. Some examples are diagnosis of Mendelian disorders^11,12^, development of precision oncology approaches for monitoring cancer evolution and the response to treatment^13,14^, and discovery of naturally occurring loss-of-function mutations that correlate with medically desirable traits to guide drug development^15,16^. Capture assays have also been used for recovery of gene transcripts in degraded archival samples that are not amenable to poly(A) selection^17^. Mitochondrial and nuclear genes share homology, so it is also possible to assemble the mitochondrial genome using off-target sequences captured during whole-exome enrichment^18^. This at the same time reflects the limited specificity of capture systems currently used. These methods have also been used for genotyping of bacteria in natural environments^19^, bacterial transcriptome profiling during host cell infection^20^, enrichment of viromes and individual viruses^21–24^, genotyping of flowering-time genes in morphotypes of oilseed rape^25^, disentangling of octoploid strawberry subgenomes^26^, identification of insecticide-resistance mutations from the mosquito genome^27^, and mapping of resistant NB-LRR loci in *Solanum*^28^.

However, all commonly used capture systems have a limited recovery rate for low abundance target sequences. Commercial streptavidin-coupled magnetic beads, in particular, are surprisingly inefficient^21,23,29^. Although they provide some enrichment of the target molecules, non-specific binding to human genomic (or non-human metagenomic) background sequences can overwhelm the low abundance target sequences. To overcome this limitation, we designed a novel capture system to minimize non-specific interaction with nucleic acids, which uses a magnetic iron oxide nanoparticle (IONP) coated with an inert silica layer. Silica coating protects the magnetic core and prepares the surface of the nanoparticle for subsequent functionalization. To attach DNA (or alternatively RNA), a layer of azide functionalized silane is deposited on the silica shell, after which the nucleic acid probes are attached with a click chemistry reaction using Cu[I]-catalyzed azide-alkyne Huisgen cycloadditions (CuAAC), which gives stable and rigid triazole linkages. Reaction conditions are designed to optimize the density of probes on the surface, further reducing the likelihood of non-specific interactions through negative charges and steric hindrance^30–33^. Performance of this novel capture system, relative to commercial streptavidin-coupled magnetic beads, is demonstrated on simulated and real clinical samples containing the hepatitis C virus (HCV) at varying concentrations.

## Results

In most capture protocols, target sequences are hybridized to biotin-labeled probes in solution, and only after that are streptavidin-coated beads used to “pull down” on-target sequences by virtue of the high affinity of streptavidin for biotin. However, we do the reverse. Our probes are conjugated to the beads prior to hybridizing with the target sequences. We do not believe this difference has any meaningful impact on capture performance, but it is something to note as it is not the conventional protocol.

### InBeads preparation and characterization

**Figure 1:**
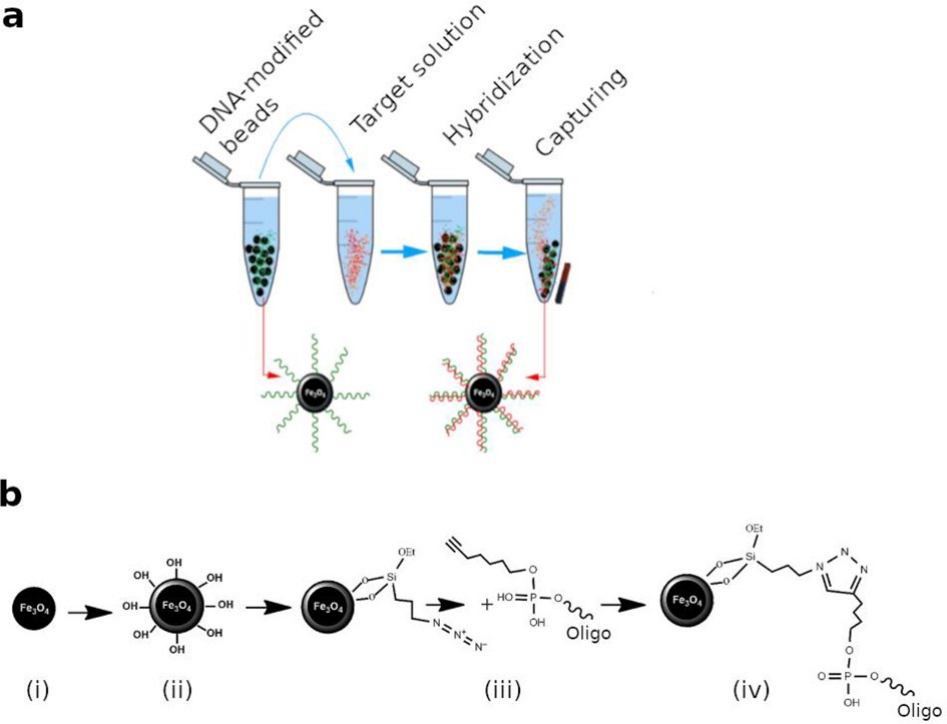
Description of InBeads capture system. (**a**) DNA-conjugated IONPs are incubated with a target solution to promote hybridization. Non-specifically hybridized DNA/RNA are removed by washes. Specifically hybridized DNA/RNA are retained on the magnetically immobilized beads. (**b**) Preparation of InBeads. (i) IONPs synthesis, (ii) silica coating, (iii) azide functionalization, and (iv) conjugation with DNA probes through click chemistry.

Iron oxide nanoparticles are silica-coated to provide an inert surface that reduces off-target hybridization (Fig. 1a). They are prepared by a solvothermal reaction, followed by silica coating, azide functionalization, and conjugation of DNA probes with click chemistry (Fig. 1b). Click chemistry was implemented with a 5′-hexynyl modification that introduces a 5′-terminal alkyne group into the DNA. This readily reacts with azide in the presence of copper, forming stable 1,2,3-triazole bonds. Cutler et al.^34^ used a different click chemistry reaction to conjugate DNA to iron oxide nanoparticles and tested for cellular uptake. They used 10 nm aminated iron oxide nanoparticles (Ocean Nanotech) coated with an amphiphilic polymer, resulting in a high cellular uptake. However, polymer coatings are prone to non-specific interactions with biomolecules, which limits their applicability for bead-based capture. We chose a silica shell precisely to reduce non-specific interactions. Our magnetic iron-oxide-nanoparticles (IONPs) also have an average diameter of 235 ± 20 nm (Fig. S1a-b), with an additional 40 nm shell for the silica coating (Fig. S1c-d). IONPs conjugated to DNA are hereafter referred to as InBeads.

To confirm that probe-target hybridization occurred, we used 20-bp 3′-fluorescein-labeled 5′-hexynyl oligonucleotides (green-oligos) and 5′-Cy5-labeled complementary oligonucleotides (red-oligos). Click chemistry was performed to conjugate azide-functionalized IONPs to the green-oligos. Hybridization against the red-oligos was promoted by heating the reaction to 65 °C and slowly cooling to room temperature. Green fluorescence (from the fluorescein-labeled probes; Fig. S2b) and red fluorescence (from the Cy5-labeled targets; Fig. S2c) could be detected before and after hybridization, respectively. This procedure was repeated using eight 90-bp probes complementary to several regions of the HCV genome. X-ray photoelectron spectroscopy (XPS) revealed that DNA grafting was successful (Fig. S2d, peaks N1s and P2p).

To optimize how DNA is immobilized on the beads surface, we performed the click-chemistry reaction under different conditions. Amount of free DNA in solution before and after the reaction was quantified with the Quant-iT OliGreen ssDNA Assay. The difference was interpreted as the amount of immobilized DNA (iDNA). Reaction conditions were assessed with probes (C and F) that bind to different regions of the HCV genome. Figure S3a shows the amount of iDNA at different concentrations of DNA in the reaction (0.1, 1, 2.4, 5, and 7.5 μM). Both probes showed an increase in iDNA with increases in DNA concentration. Yields peaked at 2.4 μM with both probes (Fig. S3a), arguing that the immobilization was most efficient at this DNA concentration. Amount of iDNA increased over time (Fig. S3b). Finally, while no DNA was immobilized on our beads without Cu(I), the reaction reached a saturation point at around 0.5 mM of Cu(I) due to the steric hindrance between DNA strands (Fig. S3c).

### Demonstrations of InBeads performance

#### Enrichment for targets and reduction in non-specific capture

**Figure 2:**
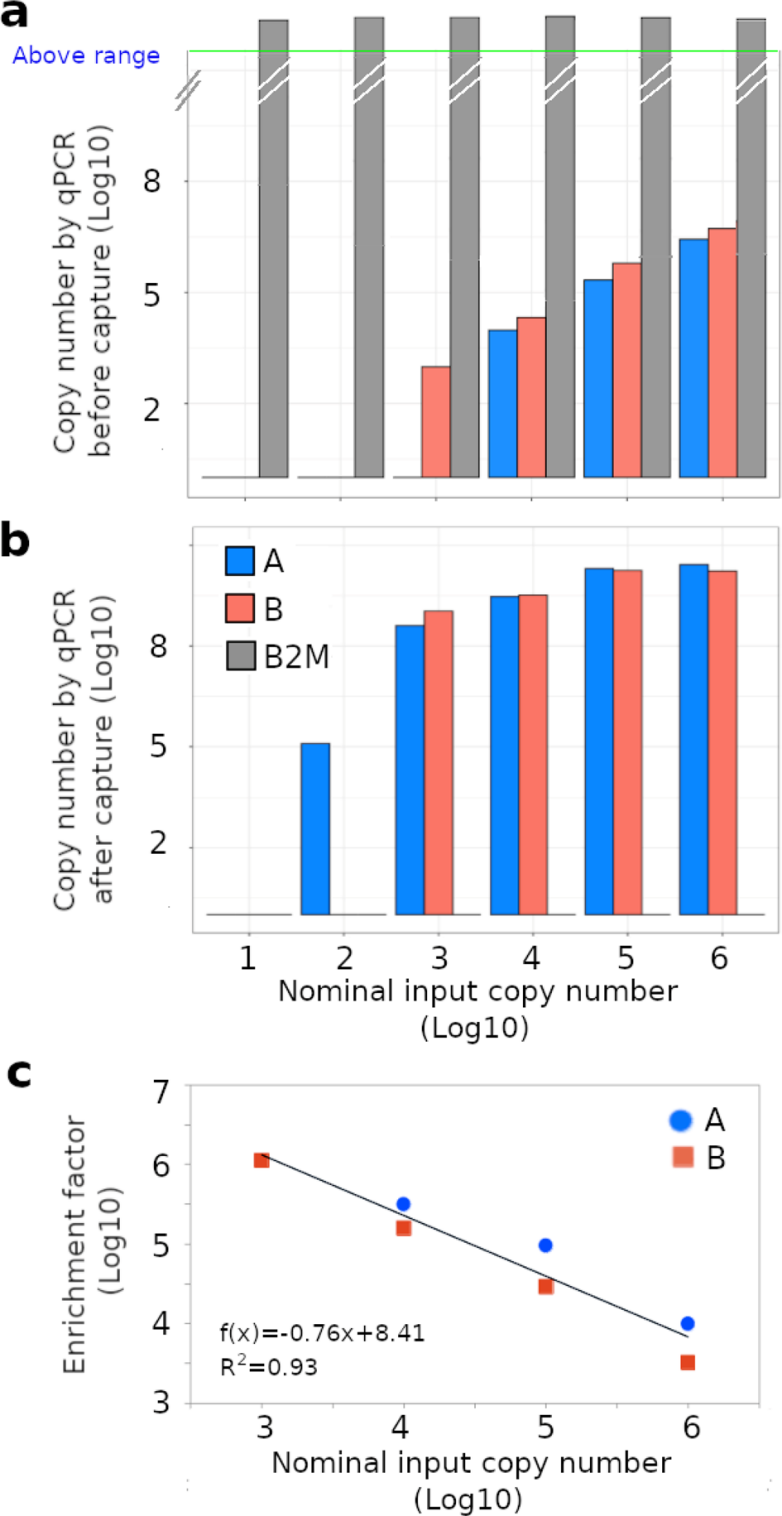
InBeads evaluation with synthetic HCV targets. Capture specificity was evaluated with a synthetic DNA gblock representing two adjacent regions of the HCV genome (A and B), mixed with 100 ng of human genomic DNA to simulate a clinical sample background. Copy number was measured by real-time qPCR, for probes A and B as well as for a human housekeeping gene B2M, both before (**a**) and after (**b**) capture. Notably, B2M copy numbers were above-range before capture and undetectable after capture. (**c**) Enrichment factor is the ratio of copy numbers before and after capture.

The hepatitis C virus (HCV) is a well-studied pathogen that causes liver inflammation, cirrhosis, and cancer. In the early stages of infection, most patients are asymptomatic, but approximately half of infected subjects develop a chronic infection. The disease often remains undiagnosed until serious liver damage has occured^35^. To evaluate the efficiency of our capture system, two sets of InBeads with probes for two adjacent regions of the HCV genome (labeled A and B) were prepared. The targets used were synthetic genomic DNAs (a single fragment of length 270 bp covering A and B), which we called gblocks and incorporated into Illumina TruSeq libraries. In parallel, a library from human genomic DNA was also constructed. A ten-fold dilution series containing 10 to 10^6^ copies of the HCV gblocks library was prepared, each mixed with 100 ng of the human library. This simulated a DNA mixture extracted from human cells infected with relatively small amounts of HCV. We used real time qPCR to measure absolute abundances (copy numbers), before and after capture, with the HCV probes A and B, and a probe for the human housekeeping gene B2M. Before capture, B2M copy numbers were out of range (Fig. 2a), while the HCV gblocks could only be detected in dilutions containing at least 10^4^ or 10^3^ molecules, using probes A or B, respectively (Fig. 2a). After capture however, recovery of the HCV gblocks was substantially enhanced (Fig. 2b,c). Stable target signals were obtained in dilutions containing at least 10^2^ or 10^3^ molecules, using probes A or B, respectively. Importantly, although B2M copy numbers were out of range before capture (Fig. 2a), they were undetectable in all instances after capture (Fig. 2b), showing the huge reduction in off-target effects associated with our system.

#### Comparison of InBeads versus streptavidin-based capture

**Figure 3:**
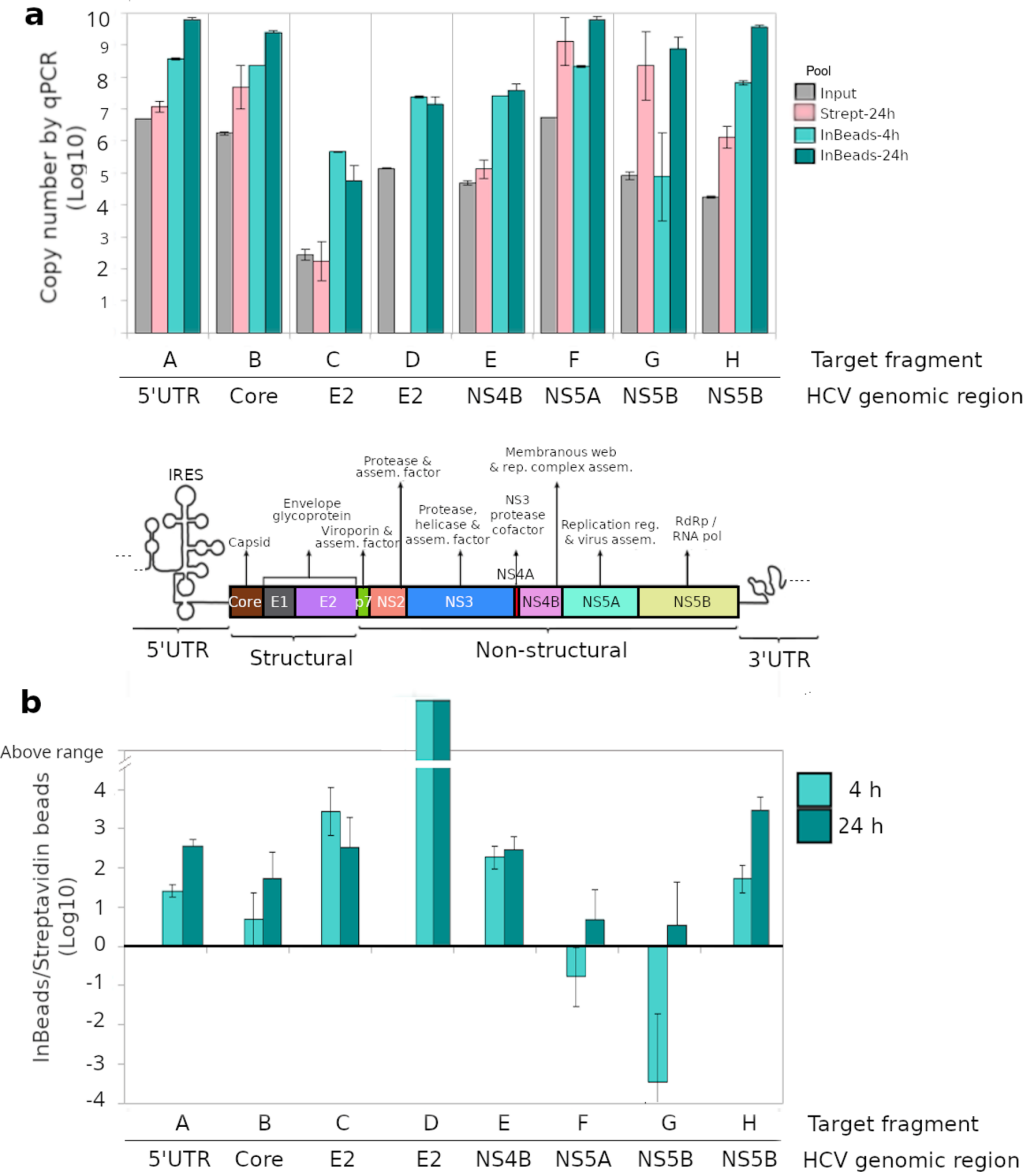
Comparing InBeads with streptavidin beads. Eight regions of the HCV genome (labels A to H) were targeted on one HCV patient (Pt339-1) sample. For InBeads, 4 h and 24 h hybridization times were used. For streptavidin, only 24 h was used. The capture experiments were done 1, 2, and 3 times for InBeads-4 h, InBeads-24 h, and streptavidin-24 h, respectively. (**a**) The copy numbers before and after capture were measured by qPCR, twice for each capture experiment. Data averaging was done on the log transformed values, i.e. geometric means and not arithmetic means. A schematic of the HCV genome shows which genes are in the targeted regions. (**b**) Ratio of InBeads to streptavidin performance. At 24 h, InBeads outperformed streptavidin for all regions, especially for D where streptavidin failed. Even at reduced 4 h hybridization, InBeads outperformed streptavidin in all but two regions (where curiously the error bars seemed larger than other regions).

To compare the performance of InBeads with streptavidin-conjugated beads (Dynabeads MyOne Streptavidin T1), eight sets of capture probes (A-H) spanning the HCV genome were designed and synthesized. These included the 5′ untranslated region (UTR) and regions encoding the core (coat) protein, the envelope glycoprotein E2, and three non-structural proteins associated with viral replication (NS4B, NS5A and NS5B). NGS libraries were constructed for an HCV-positive patient (Pt339-1) showing the highest viral load for all our patient samples, with an estimated titer of 5.6 × 10^6^ RNA genomes per ml of plasma. Results for both capture systems were variable over different HCV regions (Fig. 3a). At 24 h of hybridization, InBeads were on average 154X more effective than Dynabeads, most notably for regions A, C, D, E and H, corresponding to 5′UTR, E2, E2, NS4B and NS5B, respectively (Fig. 3b). We also developed a fast InBeads protocol, requiring only 4 h of hybridization. There was some loss in performance (Fig. 3b), but on average it was still 12.5X more effective than Dynabeads. Importantly, the Dynabeads system failed to detect region D, so the improvement is shown as ‘out of range′. For our target averaged improvements, we could not use infinity for region D, and so we adopted the best number from the other target regions. As such, the actual improvements are better than what we have indicated.

It should also be noted that qPCR saturates at copy numbers of roughly 10^9^ or 10^10^. However, the relationship between the measured and the actual copy number is monotonic. So, if one signal is larger than another, it will remain larger when qPCR measurements are compared. Moreover, the actual ratio of these two signals will be larger than what is measured through qPCR. As such, the results described here are valid despite the potential for saturation, and quantitatively even better than what we have indicated.

#### InBeads capture of HCV from plasma of infected patients

**Figure 4:**
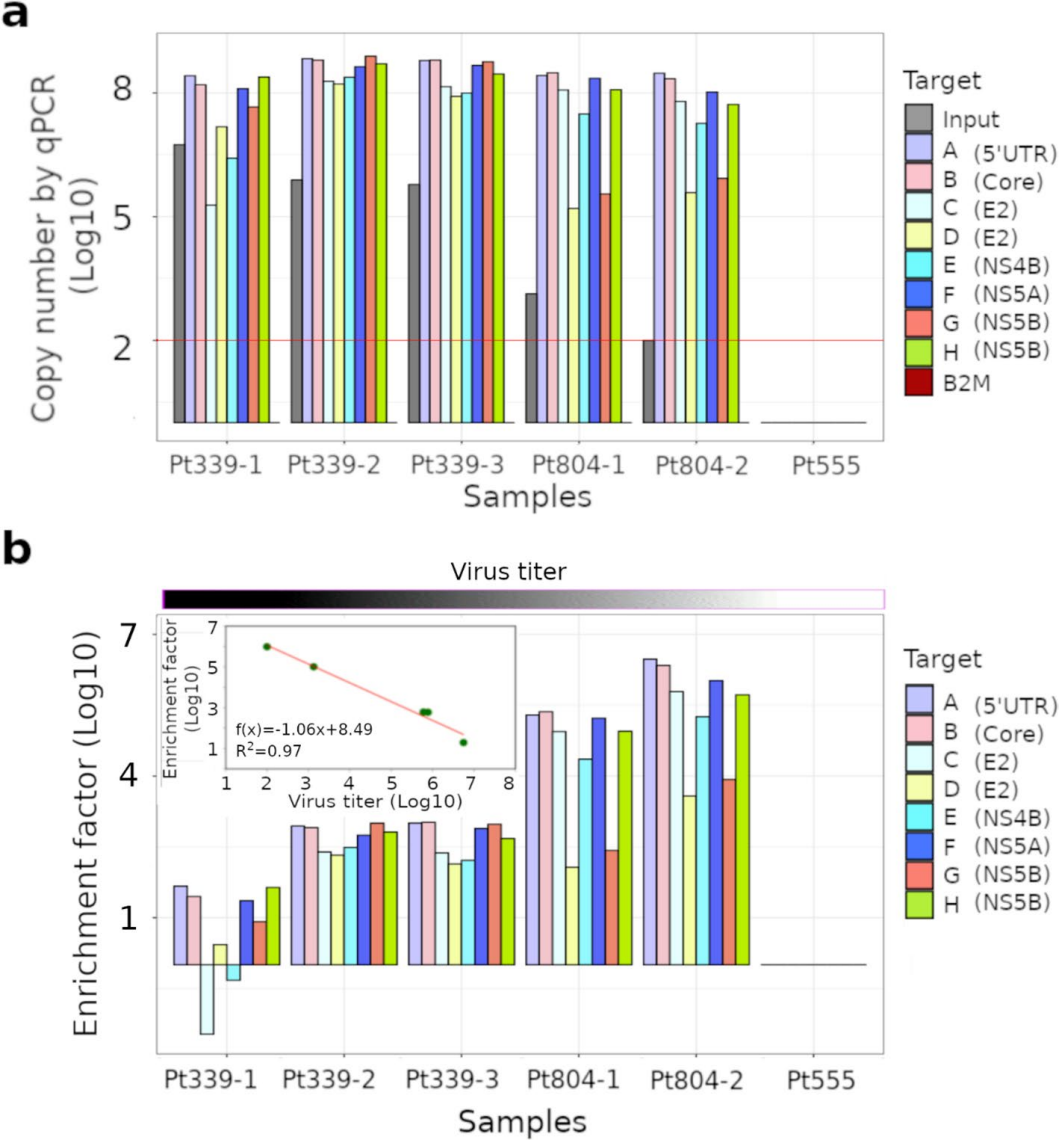
Evaluation on plasma of HCV-infected patients. The same 8 regions from Fig. 3 were targeted on five HCV patient samples and one negative control. (**a**) Measured copy numbers were zero for all target regions in the HCV-negative (Pt555) sample, and the B2M gene in all HCV-positive samples. Red line indicates the detection limit of qPCR. (**b**) Enrichment factor is the ratio of copy numbers before and after capture. This is shown as a function of sample (main plot) and viral titer (inset). One sample (Pt804-2) was below the qPCR limits of detection, but known to be HCV-positive because the patient had a relapse. Nonetheless, InBeads capture was able to detect all 8 regions.

We assessed InBeads performance on plasma samples from HCV patients, with viral titers starting at below 100 copies and ending at nearly 10^7^ copies per ml. The fast (4 h hybridization) protocol was utilized. Despite its reduced performance, relative to our 24 h protocol, we still observed consistently stable signals from all 8 HCV target fragments (Fig. 4a), even for the one sample (Pt804-2) where HCV was not detectable by qPCR but where we knew the virus was nonetheless present as the patient eventually suffered a relapse. Reassuringly, we did not find a HCV-like signal in our negative control (Pt555), and we could not detect B2M in any of our samples (Fig. 4a). Enrichment factors varied with the target region but the probe-averaged trend versus target abundance was remarkably similar to what was seen in Fig. 2. Sample Pt339-1, with the highest viral titer in this data set, had an enrichment factor of 19x (Fig. 4b). Conversely for sample Pt804-2, where we arbitrarily set the copy number to 100 as the virus was below qPCR limits of detection, the enrichment factor was 9.5 × 10^5^ (Fig. 4b).

## Discussion

There are many ways to improve a capture system. First, sensitivity can be increased to handle samples with ultra-low target sequence abundances. Second, off-target hybridization can be reduced to increase the amount of useful sequence recovered. Third, hybridization times can be minimized without overly compromising the above considerations. InBeads excelled in all aspects: sensitivity, specificity, and speed.

Our initial experiments demonstrated that InBeads exhibit very high specificity. Before capture, copy numbers for the B2M housekeeping gene were out of range for qPCR, but copy numbers became undetectable after InBeads capture with two probes complementary to the HCV genome. In contrast for conventional biotin-streptavidin systems, off-target effects are evidenced by the fact that mitochondrial genomes can be recovered in off-target reads from nuclear whole-exome capture assays^18,36^. The superiority of InBeads over the commercial capture assay (Dynabeads MyOne Streptavidin T1) was evident across all target regions when both used 24 h hybridization. When the hybridization time was reduced to 4 h for InBeads, it still outperformed Dynabeads (over 6 of the 8 HCV fragments) despite the advantage of a 24 h hybridization for the latter. On average, performance of InBeads at 24 h or 4 h of hybridization was at least 154 or 12.5-fold superior to Dynabeads at the full 24 h hybridization time, respectively. As turnaround times are often critical in clinical applications^37,38^, this difference has practical implications.

InBeads sensitivity and specificity with only 4 h hybridization was evaluated in plasma from patients with different titers of HCV. We observed a strong and consistent signal even in the one sample where HCV abundance was below the limits of detection for qPCR. Conversely, no signal was detected for the B2M human housekeeping gene. In a comparative study for several commercial capture systems, Thomson reported HCV enrichments in the 2 to 168-fold range, when compared to metagenomics sequencing^39^. Bonsall observed enrichments of up to 1000-fold for an IDT biotinylated probe capture array^40^. Briese used a whole-virome capture array and reported enrichments of 100 to 10,000-fold in comparisons with conventional viral enrichment procedures using filtration^21^. InBeads showed the highest enrichment of all, especially at the lowest viral titer, where it almost reached 10^6^.

Beyond the demonstrated superiority of InBeads for capture hybridization, which is of particular importance for detection of low abundance pathogens, we would suggest that it may be time to rethink the near-universal use of streptavidin–biotin interactions in biomedical assays. Biologists like to use biomolecules because that is what they are comfortable with, but there have been many advances in nanotechnology and inorganic chemistry. Only by collaborating with people outside our comfort zones will the field move forward.

## Materials and methods

### Synthesis of superparamagnetic IONPs (Fe_3_O_4_)

Superparamagnetic iron oxide nanoparticles were synthesized as per Tian et al^41^, with some modifications. The precursor solution was prepared by dissolving iron chloride (FeCl_3._6H_2_O; Sigma-Aldrich) in ethylene glycol (EG; Sigma-Aldrich). NaAc (Sigma-Aldrich) was added as a pH adjuster, under stirring for 20 min. The resultant solution was autoclaved at 200 ˚C for 8 h. After allowing for cooling, the black product was collected using a magnet and washed 3X with water and ethanol. The washed product was freeze-dried.

### Adding a silica coating to the IONPs (Fe_3_O_4_@SiO_2_)

IONPs were coated with a ~ 40 nm silica shell through a hydrolysis-condensation reaction of tetraethyl orthosilicate in a sol–gel process^42^. Briefly, 1 mg/ml of IONPs suspension was prepared in a solution of water, ethanol, and ammonium hydroxide (Sigma-Aldrich). The mixed solution was sonicated to produce well-dispersed magnetic particles. During sonication, 0.05 mg TEOS (Sigma-Aldrich) was added dropwise, to promote reaction with water and initiate the coating process. The reaction was continued under vigorous stirring for 6 h. Finally, the silica-coated IONPs were separated from the solution with a small magnet and freeze-dried.

### Azide functionalization of IONPs (Fe_3_O_4_@SiO_2_@N_3_)

Azide functionalization groups were added to the silica-coated surface through a 3-azidopropyl triethoxysilane deposition^43^. Following a typical protocol, 5 mg of dried silica iron oxide nanoparticles were suspended in tetrahydrofuran (THF; Sigma-Aldrich) under N_2_ protection, followed by addition of 4 mg 3-azidopropyl triethoxysilane under continuous stirring. This mixture was kept stirred two days at room temperature. Functionalized IONPs were collected by magnetic separation and then washed 3X with THF and ethanol to remove surface residues. Once again, the washed product was freeze dried.

### Attaching DNA by click chemistry (Fe_3_O_4_@SiO_2_@DNA)

DNA-probe-clicked IONPs were prepared via a copper(I)-catalyzed azide–alkyne cyclo-addition (CuAAC) reaction employing azide-functionalized IONPs and alkyne-modified oligonucleotides. Briefly, in a 500 µL test tube, 10 µL of 24 µM 5′-hexynyl modified DNA probe (IDT) in water was mixed with 3 µL of a 2 M triethylammonium acetate buffer (pH 7.0) and 10 µL DMSO. We then added 10 µL of 9.6 mg/mL azide functionalized IONPs in DMSO and water (1:1) as solvent and 3 µL of 5 mM ascorbic acid in water, after which we bubbled the reaction with argon gas for 30 s. 3.5 µL of 10 mM Cu(II)-TBTA solution in 55% DMSO (Lumiprobe) was added under argon gas protection. The reaction was incubated at room temperature overnight under rotation. The next day, the supernatant was removed, and particles were resuspended in water. This was repeated five times to eliminate residual reagents. DNA probe-clicked IONPs in water were stored at 4 ˚C and found to be stable for a year.

### Nanoparticle characterization procedures

To determine the density of DNA probes on the IONPs surface, the reaction was conducted as above, and the supernatant as well as three previous washes were collected right after the reaction was completed. DNA concentration was estimated using Quant-iT OliGreen ssDNA Assay Kit (Thermo Fisher Scientific).

IONP size and morphology were determined by field emission high- resolution scanning electron microscopy (SEM) with a Hitachi- S4800 HR instrument set at 30 kV and by transmission electron microscopy (TEM) imaging with a JEOL TEM-2200FS instrument. Samples were prepared by dropping the nanoparticle suspension on a 400-mesh carbon grid and drying it in a vacuum oven for 2 h. X-ray powder diffraction (XRD) patterns were collected using a Rigaku XRD Ultima IV instrument to study the structural properties of IONPs. X-ray photoelectron spectra (XPS) were taken on a Kratos AXIS 165 electron spectrometer with 150 W monochromatized A1 Kα radiation (1486.6 eV), whereby all peaks were referred to the signature C1s peak for adventitious carbon at 284.8 eV.

### Plasma samples from HCV patients (and controls)

Plasma samples in this study were collected from: (1) patient Pt804 (two time-points), positive for both HCC (Hepatocellular carcinoma) and HCV (Hepatitis C virus); (2) patient Pt339 (three time-points), positive for HCV; (3) a normal individual (Pt555). Viral copy numbers were determined by a procedure described in Steenbergen et al. 2010^44^. The Ethics Committee for the University of Alberta approved this investigation. Informed consent was obtained, and all experiments were performed in accordance with relevant guidelines/regulations.

### RNA extraction, reverse transcription, and library construction

Total RNA was extracted with QIAamp viral RNA kit (Qiagen) from 1 mL plasma and cleaned up by RNeasy Mini kit with RNase-free DNase set (Qiagen). This was reverse transcribed to cDNA with Superscript II Reverse Transcriptase (Thermo Fisher Scientific) and random primers following standard protocols. Second strand cDNA was synthesized with a reaction mix (Tris, pH 7.8; 50 mM MgCl2; dNTP 10 mM; DTT 0.1 M; RNase H 2U/μL; DNA Polymerase I 10U/μL) at 16 °C for 2.5 h. Double-stranded (ds) cDNA was cleaned up by QIAquick PCR Purification kit (Qiagen) and eluted in 40μL ultrapure water (Gibco). The ds cDNA was sheared on a Covaris-S2 to an average size of 400 bp, followed by library construction with NEBNext Ultra II DNA library prep kit (NEB). HCV copy numbers were measured with TaqMan qPCR according to standard protocols.

### Design of probes and dsDNA target sequences

Eight probes for different HCV regions (A to H) were selected to evaluate our InBeads against a commercial capture assay (Dynabeads MyOne streptavidin T1; Thermo Fisher Scientific). These 90-bp probe sequences (Tab. S1) were designed from the reference genome sequence (gi: 22,129,792) for HCV genotype 1 (excluding the repetitive U3 region) under the following criteria: (1) melting temperature 15-25C higher than the 65C hybridization temperature, i.e. 80-90C; (2) GC content within 40% to 65%; (3) no significant similarity to the human genome sequence as assessed by blastn; (4) no stable secondary structure (ΔG value higher than –9.0 kcal/mole) under hybridization conditions. We synthesized two sets of these 8 probes (IDT); one was modified with 5'-hexynyl (for InBeads use) and the other with 5'-biotin (for Dynabeads use). In addition, 270-bp dsDNA of HCV sequences (gblocks, covering both A and B in a single fragment) (Tab. S1) were synthesized by IDT and ligated with Illumina adaptors to be used as target fragments. These target fragments were serially diluted from 10^6^ to 10 copies and supplemented with 100 ng of a non-HCV human DNA library.

### Target sequence enrichment using InBeads

100 ng of library DNA were mixed with blocking reagents (2.5 μg of human Cot-1 DNA, 2.5 μg salmon sperm DNA, 300 pmol blocking oligos complementary to library adapter sequences) and denatured at 95 ℃ for 5 min, then maintained at 65 ℃. Hybridization buffer (10X SSPE, 10X Denhardt's solution, 10 mM EDTA, 0.2% SDS and 20U RNase Block Ribonuclease inhibitor) was freshly prepared and mixed with InBeads conjugated to equimolar amounts of each probe (20μL InBeads from stock solution, storage solution removed before use), pre-warmed to 65 ℃ and transferred to the DNA library mix to create the hybridization mix. The final volume of the hybridization mix was 30 μL, which was then incubated at 65 ℃ for 24 h. The supernatant was removed and InBeads were washed 3X with buffer (0.1X SSC, 0.1% SDS) at 65 ℃ for 10 min each. Finally, InBeads were re-suspended in 30 μL ultra-pure water.

### Target sequence enrichment by streptavidin beads

The pre-capture library mix (final volume 9 µL) was prepared by combining 100 ng DNA library with blocking reagents and denatured at 95 ℃ for 5 min, then maintained at 65 ℃. Capture probe mix was prepared by mixing the 500 ng biotin-labeled probe set (pool of eight 5'-biotin-labeled probes at equal concentrations) with 20U RNase Block Ribonuclease Inhibitor and ultra-pure water to a final volume of 7 µL, pre-warmed to 65 ℃ for at least 2 min. Hybridization buffer (10X SSPE, 10X Denhardt's solution, 10 mM EDTA, 0.2% SDS and 20U RNase block) was freshly prepared and pre-warmed at 65 ℃ for at least 5 min. 13 μL hybridization buffer and 7 µL capture probe mix were rapidly added to the pre-capture library mix to make the hybridization mix in a final volume of 29 μL, and kept at 65 ℃ for 24 h. 50 μL of Dynabeads MyOne streptavidin T1 beads (Thermo Fisher Scientific) were washed twice with 200 μL binding buffer and re-suspended with 200 µL binding buffer. The hybridization mix was directly transferred to the beads solution and incubated at room temperature for 30 min under rotation, and then the supernatant was discarded. The beads were washed once with 500 μL wash buffer I (1X SSC, 0.1% SDS) at room temperature for 15 min, then 4X with 500 µL wash buffer II (0.1X SSC, 0.1% SDS) at 65 ℃ for 10 min. After removing the wash buffer, the beads were re-suspended with 30 µL of ultra-pure water.

### Post-capture qPCR following on-beads PCR

Post-capture signal detection was a two-step process. First, we did an on-beads PCR using KAPA HiFi PCR kit (KAPA Biosystems) with the following program: 98 ℃ 10 min; 98 ℃ 20 s, 58 ℃ 15 s, 72 ℃ 30 s, 35 cycles; 72 ℃ 10 min; 4 ℃ forever; universally primed off the library construct with P5 primer (5' ACACTCTTTCCCTACACGACGCTCTTCCGATCT 3') and P7 primer (5' GATCGGAAGAGCACACGTCTGAACTCCAGTC 3'). The PCR product was purified with the QIAquick PCR Purification kit (Qiagen), after which we performed a qPCR using SYBR Green PCR Mastermix (Thermo Fisher Scientific). Eight pairs of primers were designed (Tab. S1); for each pair, one primer was located in the on-target region, with the other in the flanking region, to ensure a product length in the 150–200 bp range and to avoid amplifying probe sequences. 2 µL of purified on-beads PCR product was used for each qPCR reaction in a final volume of 25 µL. Each qPCR reaction was carried out in duplicate in an ABI 3700 real-time PCR system (Applied Biosciences). The following thermocycling program was used: 95 ℃ 10 min, followed by 40 cycles of 95 ℃ 15 s, 60 ℃ 1 min. A final dissociation step was performed to determine the melting curve. The copy numbers were calculated based on a standard curve that was generated from a serially diluted plasmid with HCV genomic sequence (from 10^6^ to 10 copies per µL).

## Supplementary Information

Supplementary Information.
