# Supplementary material for: Enrichment of low abundance DNA/RNA by oligonucleotide-clicked iron oxide nanoparticles
Source: Sci Rep. 2021 Jun 22;11:13053. doi: 10.1038/s41598-021-92376-9 (PMC8219684; doi:10.1038/s41598-021-92376-9)
Supplement: Supplementary file 1 — Supplementary Information. [file 41598_2021_92376_MOESM1_ESM.docx]

**Enrichment of Low Abundance DNA/RNA by Oligonucleotide-Clicked Iron Oxide Nanoparticles**

Fereshte Damavandi^1,2,*^, Weiwei Wang^3,4,*^, Wei-Zheng Shen^1,2^, Sibel Cetinel^1,2,5^ Tracy Jordan^3^, Juan Jovel^3^, Carlo Montemagno^1,2,#^, Gane Ka-Shu Wong^3,6,**^

^1^Ingenuity Lab, 1-070C, 11421 Saskatchewan Drive NW, T6G 2M9, Edmonton AB, Canada

**^2^**Department of Chemical and Materials Engineering, University of Alberta, T6G 2V4, Edmonton, AB, Canada

^3^Department of Medicine, University of Alberta, T6G 2E1, Edmonton, AB, Canada

^4^Present address: Geneis Inc., Bldg A, 5 Guangshun North Street, Beijing, China

^5^Present address: Nanotechnology Research and Application Center (SUNUM), Sabanci University, Istanbul 34956, Turkey

^6^Department of Biological Sciences, University of Alberta, T6G 2E9, Edmonton, AB, Canada

^#^Deceased on October 11, 2018.

^*^Fereshte Damavandi and Weiwei Wang contributed equally to this work.

^**^**Corresponding Author:** E-mail: [gane@ualberta.ca](mailto:gane@ualberta.ca).

**Notes:** The authors declare that they have no conflict of interest.

**SUPPLEMENT LEGENDS**

**Figure S1. Physical properties of iron-oxide nanoparticles**. (**a**) IONPs viewed by scanning electron microscopy (SEM). (**b**) Size distribution computed by Image J software. (**c**) Silica-coated IONPs under SEM. (**d**) Transmission electron microscopy (TEM) of the core shells of silica-coated IONPs showing 40nm thickness for the silica coating.

**Figure S2. Confirmation of probe-target hybridization.** (**a**) Bright-field microscopy image of IONPs. (**b**) Fluorescence microscopy of IONPs conjugated to Alexa Fluor 488 (green) labeled DNA probes. (**c**) Fluorescence microscopy of DNA-clicked IONPs after hybridization to Cy5 (red) labeled complementary DNA. (**d**) X-ray photoelectron spectroscopy (XPS) of DNA-clicked IONPs with additional peaks at N1s and P2p. Scale bars are 20 μm.

**Figure S3. Optimization of DNA density on InBeads surface.** Left and right subpanels used probes complementary to target regions F (NS5A) and C (E2), respectively. (**a**) Saturation curve for immobilized DNA (iDNA) vs reaction DNA. Yield is the ratio of immobilized to reaction DNA.(**b**) Increase in iDNA as a function of time. (**c**) Decrease in iDNA past the saturation point of ~0.5 mM Cu(I) concentration. For subpanels (b) and (c) the reaction concentration was set to 2.4 μM, consistent with the maximum yield. Polynomial fits are guides for the eye, and nothing more.

**Table S1. Sequences for gblocks, probes, and primers.**

**
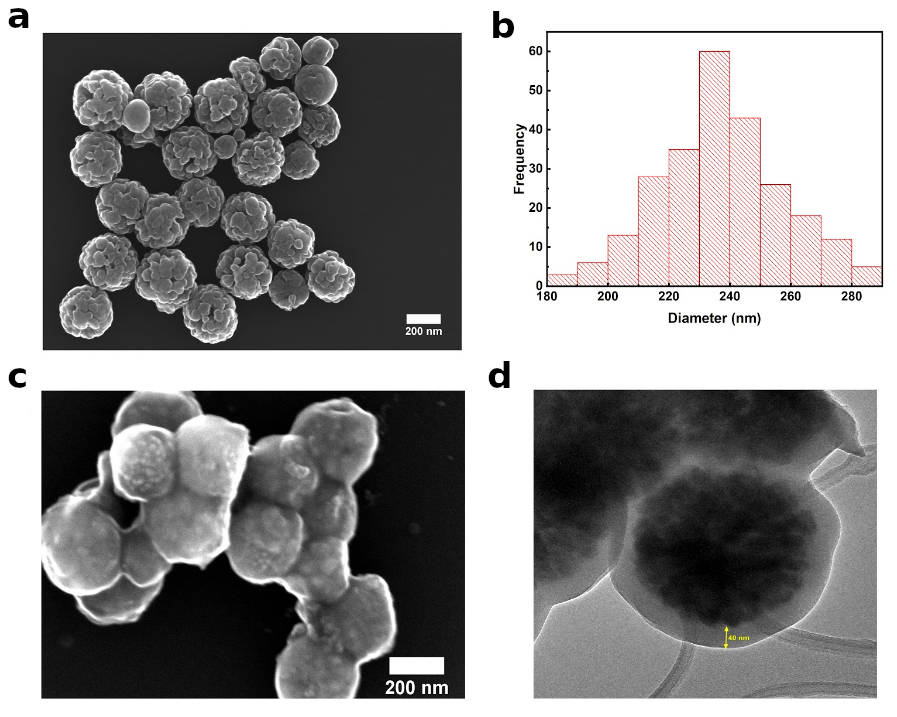
**

**Figure S1.**

**
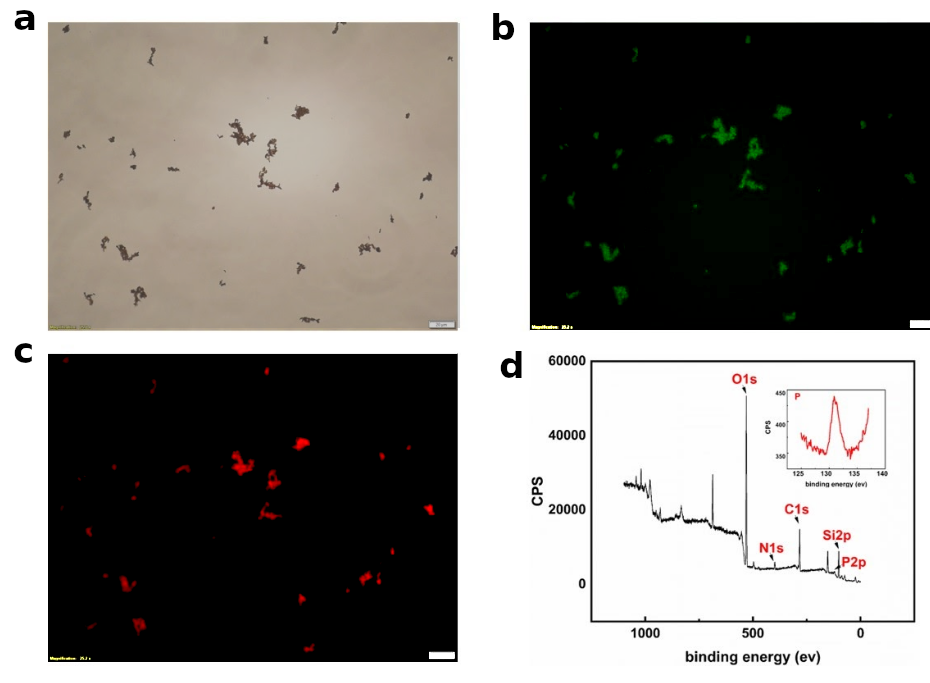
**

**Figure S2.**

**
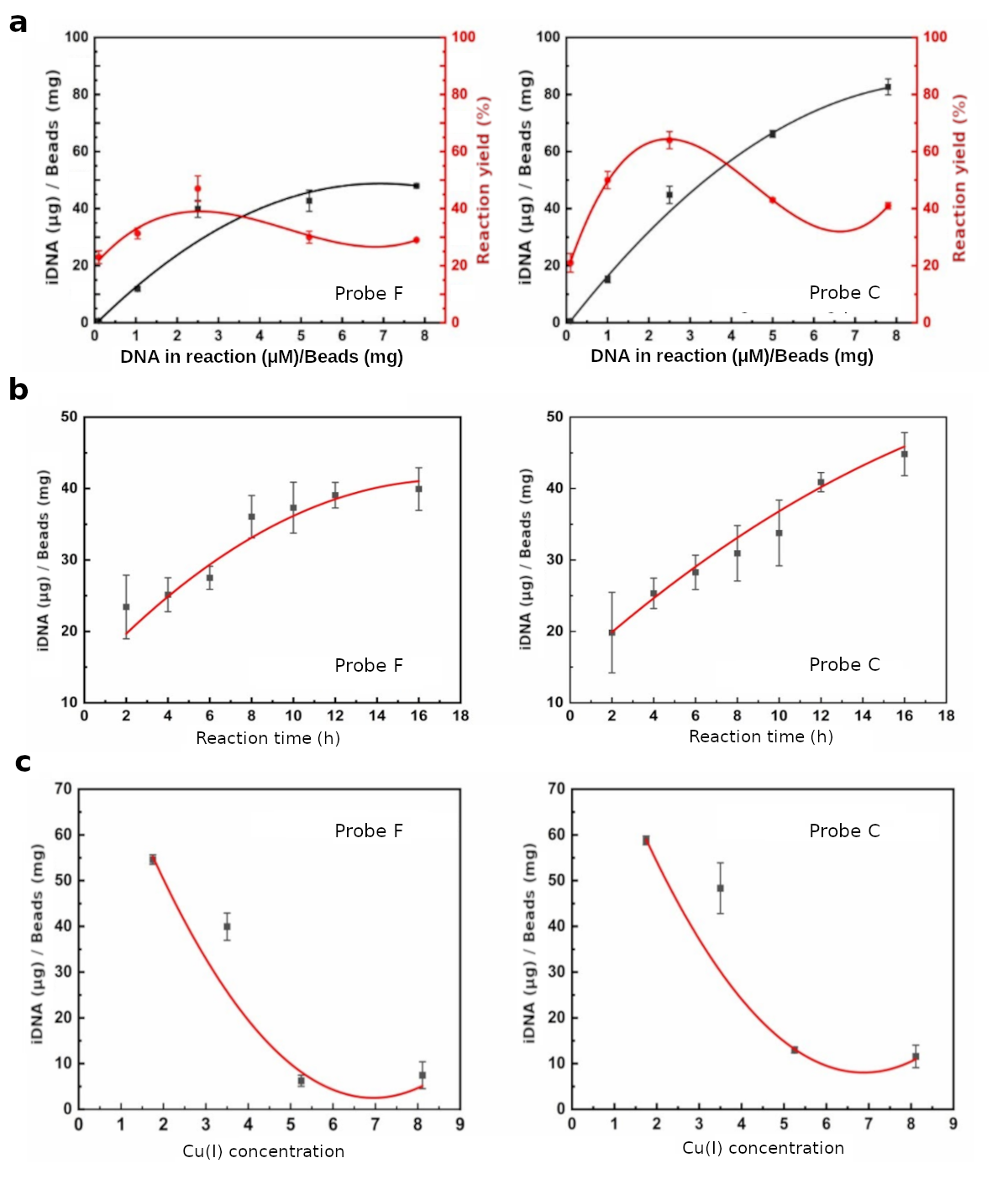
**

**Figure S3.**

**Table S1.**
